# Foot immersion with and without neck cooling reduces self-reported environmental symptoms in older adults exposed to simulated indoor overheating

**DOI:** 10.1080/23328940.2024.2394341

**Published:** 2024-09-11

**Authors:** Fergus K. O’Connor, Gregory W. McGarr, Emma R. McCourt, Robert D. Meade, Glen P. Kenny

**Affiliations:** a Human and Environmental Physiology Research Unit, School of Human Kinetics, Faculty of Health Sciences, University of Ottawa, Ottawa, Canada; b Consumer and Clinical Radiation Protection Bureau, Health Canada, Ottawa, Ontario, Canada; c Clinical Epidemiology Program, Ottawa Hospital Research Institute, Ottawa, Canada

**Keywords:** Cooling interventions, heat stress, thermoregulation, psychometric stress, heat wave

## Abstract

While foot immersion and neck cooling have been recommended for protecting heat-vulnerable groups, recent evidence does not support their efficacy for mitigating increases in physiological heat strain in older adults. However, their influence on self-reported environmental symptoms and mood-state remains unclear. Seventeen older adults (nine females, median [interquartile range] age: 72 [69–74]) completed three randomized heat exposures (6-h; 38°C, 35% relative humidity) with no cooling (control), foot immersion to mid-calf in 20°C water for the final 40-min of each hour (foot immersion), or foot immersion with a wet towel (20°C) around the neck (foot immersion with neck cooling). Core temperature, skin temperature, and heart rate areas under the curve (AUC) were assessed as indicators of cumulative physiological strain. Environmental symptom scores (68-item environmental symptoms questionnaire) and mood disturbance (40-item profile of mood states questionnaire) were evaluated at end-heating (adjusted for pre-exposure). Core temperature AUC was not different between conditions (*p* = 0.418). However, the skin temperature and heart rate AUCs were 11.8°C · h [95% confidence interval: 8.1, 15.5] and 12.5 bpm · h [0.1, 24.8] lower for foot immersion and 16.6°C · h [12.9, 20.3] and 19.6 bpm · h [7.2, 32.0] lower for foot immersion with neck cooling compared to control (*p* ≤ 0.032). Environmental symptom scores were 0.8-fold [0.6, 1.0] lower for both foot immersion with and without neck cooling, compared to control (both *p* = 0.036). Mood disturbance was not different between conditions (both *p* ≥ 0.275). Foot immersion with and without neck cooling reduces self-reported environmental symptoms in older adults despite having little effect on physiological heat strain.

## Introduction

Climate change is increasing the frequency and severity of hot weather [1] posing a growing threat to the health of older persons who are among the most vulnerable to heat [1–4]. For example, of the 619 heat-related deaths during the 2021 Western North America heat wave, 67% of decedents were over the age of 70, and 90% were over the age of 60 [5]. This is largely a result of age-related impairments in heat loss responses of skin blood flow and sweating [4], limiting the capacity for heat dissipation [6].

While the negative effects of heat on human health are well known, preventing heat illness requires that individuals take protective action (i.e. change behavior and implement cooling strategies) to mitigate the associated dangers. Having access to an air conditioner and deciding to employ it during indoor overheating is perhaps the most effective behavioral approach for reducing the health impacts of heat exposure [7]. However, older adults are less likely to have, or use an air conditioner during hot weather, which may be in part due to the high cost of operation [8,9]. Consequently, there have been increasing calls for the use of low-cost cooling modalities to protect heat vulnerable persons [8,10]. Owing to the ease of implementation, foot immersion has been recommended as a cooling intervention for older adults [8]. Advantages of foot immersion are that it does not require an external power source and therefore can be used during heatwave-related power outages [8,11] and it can be easily combined with other commonly recommended cooling interventions, such as the application of wet towels to the head or neck to enhance cooling [12].

Despite these recommendations [8], a recent study from our laboratory found that foot immersion, whether applied alone or in combination with neck cooling, led to marked reductions in core temperature and cardiovascular strain in older adults during prolonged (6-h) exposure to a simulated overheated dwelling (38°C, 35% relative humidity) [13] reflective of recent deadly heat waves in North America. However, foot immersion in conjunction with neck cooling did result in an increased perception of comfort at the completion of the 6-h heat exposure [13]. While assessing thermal comfort provides a subjective evaluation of how limb and neck cooling may influence an individuals’ overall satisfaction with their thermal environment, it does not offer any direct insights into the specific effects of elevated thermal strain during daylong heat exposure on physical symptoms or indicators of mood-state that may be associated with heat illness under these conditions. Importantly, we previously showed that mid-day cooling center use (2-h air conditioning) during a daylong heat exposure can effectively mitigate self-reported symptoms related to heat illness as well as mood disturbance in older adults after returning to the heat, in conjunction with a cumulative reduction in thermal strain [14]. However, visiting a cooling center during hot weather or a heat wave may not always be practical or preferable for some older adults and it remains unclear if similar positive effects on these perceptual indicators of thermal strain can be achieved when using commonly recommended low-cost cooling interventions such as limb immersion and neck cooling within the home throughout daylong heat exposure.

To address the above mentioned knowledge gaps, we used validated questionnaires [15,16] to assess environmental symptoms and mood state during the 6-h heat exposure with and without lower limb immersion and neck cooling. Our primary objective was to assess whether foot immersion with or without neck cooling influenced self-reported environmental symptoms and mood disturbance in older adults. We also assessed the relationship between perceptual responses and cumulative thermal strain experienced throughout the 6-h heat exposure.

## Methods

This single-site laboratory-based randomized crossover trial (NCT05601713) was approved by the University of Ottawa Health Sciences and Science Research Ethics Board (H-11-20-6234). The work was conducted in accordance with the Declaration of Helsinki. Written and informed consent was obtained from all participants prior to enrollment. Experimental procedures are described in full in the supplemental materials of the original study report [13]. Pertinent details are repeated here for context.

### Participants

Prospective participants were eligible if they were 65–85 y old, nonsmoking, English or French speaking, and were able to provide written informed consent. Exclusion criteria included severe physical restriction (e.g. due to disease, intermittent claudication, renal impairment, active proliferative retinopathy, unstable cardiac or pulmonary disease, disabling stroke, severe arthritis, etc.), use of or changes in medication judged by the investigators to make involvement in this study inadvisable, peak aerobic power exceeding the 50th percentile of age- and sex-specific normative values [17], or cardiac abnormalities identified during preliminary screening. Of 25 older adults (≥65 y) screened for eligibility, 17 participated in the trial, which ran between September 2022, and April 2023. No participants had undergone heat acclimation prior to, or during enrollment, nor were they employed in occupations where they were frequently exposed to hot environmental conditions. We chose to confine testing to the North American Fall and Winter, as many heat-related health events are pronounced early in the summer [18] when vulnerable groups are less acclimatized [19].

### Experimental procedures

On separate days, enrolled participants completed an initial screening session and three randomized experimental sessions. Participants wore light summer clothing (sandals, t-shirt, shorts: ~0.4 Clo) for all sessions, which were separated by a minimum of 4 d. The median (interquartile range) between each session was 6 (6, 7) d. Each experimental session differed only in the cooling intervention applied (described below).

### Preliminary screening

Participants were familiarized with all procedures and measurements and completed the Canadian Society for Exercise Physiology Get Active Questionnaire and the American Heart Association Pre-Participation Screening Questionnaire to assess their eligibility to participate. Thereafter, resting arterial blood pressure was measured in triplicate (~1 min between measures) via automated oscillometry (UM-211, A&D Medical) of the brachial artery in accordance with American Heart Association recommendations [20]. Descriptive anthropomorphic data were then collected. Body height and mass were determined via a stadiometer (model 2391, Detecto) and a digital weighing terminal (model CBU150X, Mettler Toledo Inc.), respectively, and used to calculate body mass index and surface area [21]. Participants then performed an incremental exercise stress test to volitional fatigue on a semi-recumbent cycle ergometer (Corival, Lode B.V., Groningen, Netherlands) while being monitored via 12-lead electrocardiogram by a certified clinical exercise physiologist. Indirect calorimetry was used to quantify peak oxygen uptake (MCD Medgraphics Ultima Series, MGC Diagnostics, MN, USA) during the exercise stress test. All preliminary screening was conducted in thermoneutral conditions (~22°C).

### Experimental protocol

Participants completed three randomized, 6-h experimental sessions. Each experimental session commenced between 06:00 and 09:00. Upon arrival to the laboratory, adequate hydration status was confirmed (urine-specific gravity of ≤1.025 [22] [TS 400 total solids refractometer, Reichert Technologies, New York, USA]). Participants then inserted a general-purpose thermocouple temperature probe for the continuous measurement of rectal temperature. Body mass (nude) was recorded before participants were transferred to a temperate room (~22°C). Participants were seated for ~30 min, while instrumentation and baseline measurements were completed (described in Outcome measurements and analysis). During this period, participants were asked a series of questions regarding heat exposure, physical activity and sleep habits, dietary intake, and general well-being in the preceding days. Participants were instructed to replicate their activity patterns as closely as possible prior to each study visit. Thereafter, participants completed a cardiac autonomic response test battery (described in detail elsewhere) [23].

Participants were then transferred to a climate-controlled chamber regulated to 38°C, simulating an overheated dwelling during a heat wave [5], to begin the 6-h heat exposure (35% relative humidity, <0.2 m/s air velocity, no radiant heat sources). The exposures differed only in the cooling intervention applied: no cooling (control), submersion of the feet to mid-calf in 20°C water for the final 40 min of each hour (foot immersion), or foot immersion with a wet towel (20°C) draped around the neck (foot immersion with neck cooling). Tap water (~20°C) was made available to participants *ad libitum*. Participants consumed a self-provided lunch (e.g. sandwich) within the first 3-h of exposure. At the end of the 6-h heat exposure period, participants completed an identical cardiac autonomic response test battery before exiting the climate chamber and recording a final nude body mass.

### Outcome measurements and analysis

#### Body core and skin temperatures

Rectal temperature was continuously monitored as an index of core temperature via a general-purpose thermocouple temperature probe (Mon-a-therm General Purpose Temperature Probe, Mallinckrodt Medical Inc.) inserted ~12–15 cm past the anal sphincter. Data were collected at a sampling rate of 1 kHz using LabChart physiological data analysis software (version 8.1.16.2019, ADInstruments) and downsampled to 5 s sampling intervals before analysis. Ingestible temperature capsules (e-Celcius, BodyCap, Caen, France) were utilized to monitor gastrointestinal temperature in three participants who refused the rectal thermocouple. The gastrointestinal temperature pills utilized have previously been shown to be valid and reliable, with a systemic difference between recorded and actual temperature <0.1°C [24,25]. Gastrointestinal temperature data were collected at 15-s intervals.

Skin temperatures were collected at 1-min intervals using temperature data loggers (DS1922L Thermochron, OnSolution Pty Ltd, Sydney, Australia) affixed to eight body regions using double-sided adhesives and medical tape. Mean skin temperature was calculated according to the weightings recommended in ISO 9886:2004: 7% forehead, 17.5% right scapula, 17.5% upper left chest, 7% upper right arm, 7% right forearm, 5% left hand, 19% right anterior thigh, and 20% left calf [26].

#### Cardiovascular responses

Heart rate and systolic and diastolic arterial blood pressures were measured pre-exposure (baseline) and at the end of each hour of exposure via an independently validated brachial artery oscillometer [27] (UM-211, A&D Medical, Tokyo, Japan) in accordance with American Heart Association guidance [20].

#### Cumulative physiological strain

In accordance with similar studies assessing physiological and perceptual responses of older adults exposed to simulated indoor overheating [14,28], the areas under the curve (AUC) for body core and mean skin temperature (both in °C · h), heart rate (bpm · h), as well as systolic and diastolic blood pressure (mmHg · h) were estimated using the “trapz” function from the “pracma” R package [29] as measures of cumulative thermal strain across the entire 6-h heat exposure. This analysis can be interpreted as taking the sum of the average elevations of each index above baseline for each hour of exposure. For example, a cumulative thermal strain of 6°C · h for body core temperature over 6-h is equivalent to an average temperature elevation above baseline of 1°C for each hour of exposure (derived from dividing the AUC by the total exposure time).

#### Perceptual responses

Subjective reactions and environmental symptoms associated with the heat exposure were determined from the Environmental Symptoms Questionnaire, version 4 (ESQ-IV). The ESQ-IV is a validated 68-item, self-administered questionnaire that has been successfully used to identify symptoms during exposure to a wide variety of environmental conditions, including heat exposure. For each equally weighted question, participants were asked to assess and describe “how you have been feeling today” using a 6-point Likert scale (0 = “Not at all,” 1 = “Slight,” 2 = “Somewhat,” 3 = “Moderate,” 4 = “Quite a bit,” or 5 = “Extreme”). Total Symptom Score was subsequently calculated by taking the sum of the intensity ratings from the 68 individual items using reverse scoring for the three positive items from the list (“I Felt Good,” “I Felt Alert,” and “I Felt Wide Awake”).

The abbreviated 40-item Profile of Mood States (POMS-40) questionnaire was used to evaluate potential mood changes in response to heat exposure. The POMS-40 is a validated, self-administered questionnaire covering seven distinct aspects of mood state across two positive subscales (Esteem-Related Affect, and Vigor) and five negative subscales (Fatigue, Tension, Confusion, Anger, and Depression), described across 40 distinct adjectives (reference). For each equally weighted question, participants were asked to describe “how you feel right now” using a 5-point Likert scale (0 = “Not at all,” 1 = “A little,” 2 = “Moderately,” 3 = “Quite a lot,” or 4 = “Extremely”). The values of items associated with a specific subscale (e.g. fatigue) were summed to calculate its score. Total Mood Disturbance (TMD) was calculated by subtracting the sum of scores for the two positive subscales from the sum of scores for the five negative subscales and then adding a constant of 100 to remove possible negative scores. As such, higher TMD scores indicate greater mood disturbance (i.e. more negative mood-state). Energy Index (EI) scores were assessed by subtracting Fatigue from Vigor, providing an index of a participant’s perceived energy levels [30]. As such, more negative EI scores indicate lower perceived energy levels, whereas more positive EI scores indicate greater perceived energy levels.

Both questionnaires were administered during pre-heating baseline and at end-heating after participants were resting quietly in the seated position for a minimum of 30–45 min. Questionnaires were administered on a touchscreen computer tablet using Survey Monkey (Momentive Global Inc., New York, USA). Written instructions were provided on screen at the beginning of each questionnaire. Participants were given as much time as needed to complete both questionnaires without interference and additional instructions from the researchers. Examples of the questionnaire interface from Survey Monkey are provided in the supplemental materials.

### Statistical analysis

#### Sample size determination

As part of a larger project [13], body core temperature was used to calculate the sample size, with a ≥ 0.2°C reduction chosen as the minimal clinically meaningful effect. It was determined that a minimum sample size of 15 participants was required to detect this effect with 84% power [13]. However, the current findings should be considered exploratory as an additional power calculation was not conducted for the assessment of self-reported environmental symptoms and mood state.

#### Physiological responses

Physiological responses were analyzed using a linear mixed model that included the cooling condition as a fixed effect (categorical predictor: no cooling (control), foot immersion, foot immersion with neck cooling). To account for repeated measurements due to the crossover design, a random intercept was modeled for each participant. Separate models were run for each index of physiological strain (area under the curve for hours 0–6 for body core temperature, mean skin temperature, heart rate, systolic blood pressure, and diastolic blood pressure).

#### Questionnaire responses

End-heating scores for total symptoms score (ESQ-IV; count data) and total mood disturbance (POMS-40; count data) were analyzed with negative binomial generalized linear mixed models using a log link function, except for energy index (POMS-40; difference score), which was analyzed with a traditional linear mixed model (identity link). The fixed effects for all models were pre-heating score (continuous predictor) and cooling condition (as above), and a random intercept was modeled for each participant.

To examine the effects of cumulative physiological strain on end-heating questionnaire scores, we fit additional mixed effects model for each outcome variable that included an index of physiological strain (physiological variable AUC) as a fixed effect as well as pre-heating score and cooling condition (as above) and a random intercept was modeled for each participant. For these analyses, a multiplicative model was fit initially (i.e. model with effects of pre-heating score, as well as the cooling condition, physiological strain index, and their interaction). Additive models were employed for cases in which the interaction term was not statistically significant at the α = 0.05 level.

For both questionnaires, we also determined the mean group ratings for all individual items at end-heating, as well as the prevalence of participants reporting scores ≥1 for each item (i.e. participants reporting at least “Slight” [1] for ESQ-IV or “A little” [1] for POMS-40). For both questionnaires, individual item responses for the foot immersion and foot immersion with neck cooling conditions were rank ordered by mean ratings and then by the prevalence of responses for the no-cooling control condition. Individual items with group mean ratings ≥1 are presented as indicators of a minimum stimulus response [16].

All traditional linear mixed models were visually examined for heteroscedasticity and normality of residuals using residual and Q-Q plots. Descriptive data are presented as mean (standard deviation) with between-condition differences presented as mean difference [95% confidence limits]. All generalized linear mixed models were visually examined for heteroscedasticity and residual diagnostics. They were also tested for zero-inflation and dispersion. Descriptive data for generalized linear mixed models are presented as mean (standard deviation). Between-condition differences are presented as fold-differences [95% confidence limits] on the response scale, which were calculated by exponentiating values from the link (log) scale. A two-sided p < 0.05 was considered statistically significant. Reported *p* values were adjusted for multiplicity using the Holm-Bonferroni procedure (all comparisons for each model were considered as a family). All statistical analyses and figures were completed using R version 3.6.1 (R Core Team [31]. https://www.R-project.org/) and associated packages [32–36].

## Results

### Participant characteristics

A total of 25 individuals were assessed for eligibility and 17 participated (9 females, median (IQR) age: 72 (69–74) y, height: 168 (159–172) cm, mass: 71.2 (55.5–77.5) Kg, body mass index: 24.8 (22.0–26.6) kg/m^2^), body surface area 1.8 (1.6–1.9) m^2^. Participant characteristics are presented in Table 1.

**Table 1.** Physical characteristics of enrolled participants.

Variable	All participants (*n* = 17)
Age, *years*	72 (69 – 74)
Sex, *No. (%)*	
Women	9 (53%)
Men	8 (47%)
Smoking status^*a*^	
Never	11 (65%)
Past	6 (35%)
Habitual physical activity, *min/week*^*b*^	180 (60 – 240)
Types of physical activity^*b*^	
Walking	13 (76%)
Jogging, biking, swimming	7 (41%)
Aerobics, floor exercises	3 (18%)
Organized sports	3 (18%)
Peak aerobic power (VO_2peak_), mL*/kg/min*^*c*^	25 (22 – 27)
ACSM VO_2peak_ percentile, *%* ^*c*^	17 (13 – 35)
Using prescribed medications, *No. (%)*^*d*^	15 (88%)
Hemoglobin A_1c_, *%*	5.6 (5.4 – 5.8)

Values are median and interquartile range (IQR) or No. participants (%).

^a^Smoking status determined via participant self-report. Prospective participants were excluded if they were currently smoking. All past smokers quit ≥19 y prior to participation.

^b^Participant self-reported physical activity level determined via the Canadian Society for Exercise Physiology Get Active Questionnaire (GAQ). The types of physical activity performed were determined via the Kohl Physical Activity Questionnaire.

^**c**^Prospective participants peak aerobic power (VO_2peak_) was assessed during an incremental cycling test to volitional fatigue. Volunteers were excluded if their VO_2peak_ exceeded the 50^th^ percentile of age- and sex-specific normative data published by the American College of Sports Medicine (ACSM).

d Participant prescription medication use determined via self-report. Some participants reported medications that have been suggested to increase heat vulnerability (e.g. antidepressants) or are taken to treat health conditions known to reduce heat tolerance (e.g. type 2 diabetes, heart conditions) [4]. Reported medications included: antihypertensives (e.g. ACE inhibitors, angiotensin II receptor blockers, diuretics, calcium channel blockers; *n* = 4), antidepressants (e.g. serotonin and norepinephrine reuptake inhibitors, selective serotonin reuptake inhibitors, *n* = 3), metformin (*n* = 2), statins (*n* = 6), topical creams/ointments (*n* = 1), hormonal replacements (*n* = 2), corticosteroids (*n* = 2), bronchodilators (*n* = 2), antihistamines (*n* = 1), and medications treating gastrointestinal reflux (*n* = 2), hypoactive thyroid (*n* = 2), benign prostatic hyperplasia (*n* = 2), occasional pain (e.g. for headaches and joint pain; *n* = 2), osteoporosis (*n* = 2), gout (*n* = 1), hair loss (*n* = 1), antibiotics (*n* = 1), and sodium-glucose cotransporter inhibitors (e.g. blood sugar management; *n* = 1).

### Environmental symptoms

**Figure 1.**
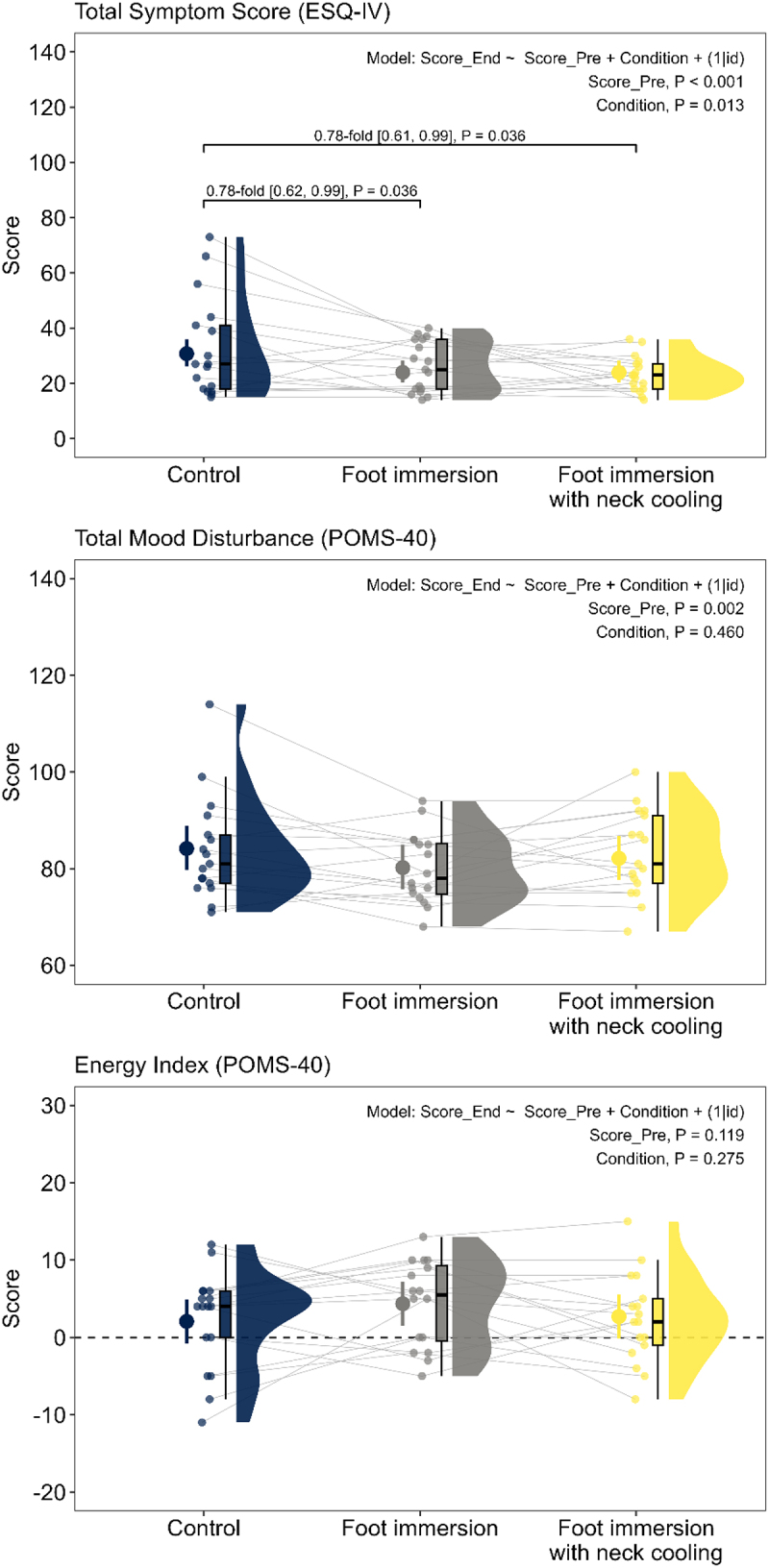
Influence of cooling condition on the perceptual responses for older adults (69-74 y; n = 17) at the end of three randomized 6-h extreme heat exposures (38°C, 35% relative humidity). Conditions differed only in the applied cooling intervention: no cooling (control), submersion of the feet to mid-calf in 20°C water for the last 40 min of each hour (foot immersion), or foot immersion with a wet towel (20°C) draped around the neck (foot immersion with neck cooling). Total symptom score (top panel) was derived from the 68-item environmental symptoms questionnaire, version 4 (ESQ-IV). Total mood disturbance (middle panel) and Energy Index (lower panel) were both derived from the 40-item profile of mood states questionnaire (POMS-40). Data are presented as modeled means (large points) and 95% confidence intervals (error bars) with individual scores (small points), boxplots (median and interquartile range), and density plots (distribution) for each condition. End-heating symptom scores were evaluated using a negative binomial generalized linear mixed model with a log link function, except for energy index (POMS-40; difference score) which was analyzed with a traditional linear model (identity link). Cooling condition (categorical predictor), as well as pre-exposure score (Score_pre; continuous predictor) were included as fixed effects. To account for repeated measurements due to the crossover design, a random intercept was modeled for each participant. Between-condition contrasts are presented as fold-differences [95% confidence limits] for total symptom score and total mood disturbance after back transformation from the log scale and as mean differences [95% confidence limits] for Energy Index. Significance was set at p < 0.05 (two-sided). *p* values were adjusted for multiplicity using the Holm-Bonferroni procedure.

**Figure 2.**
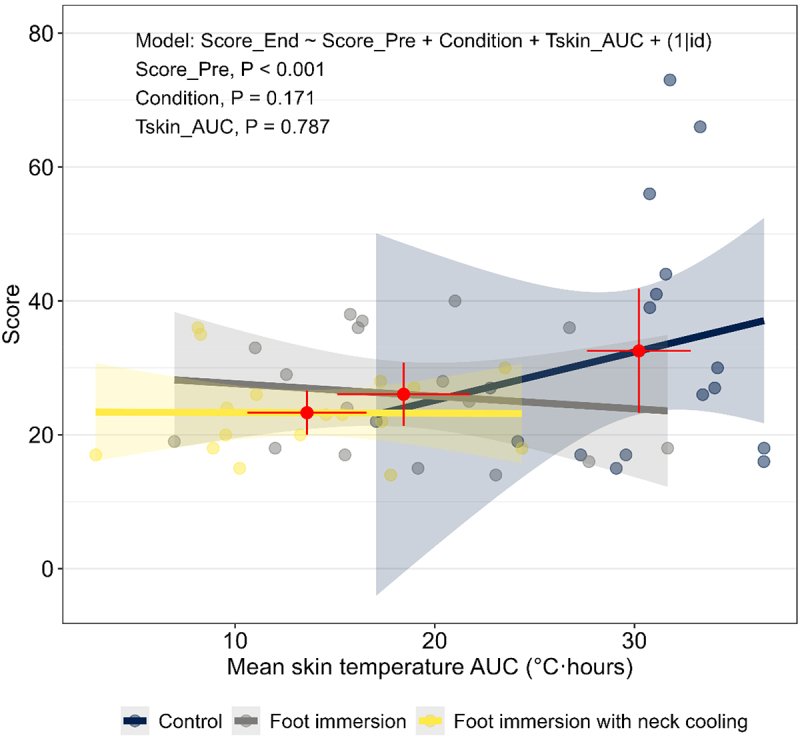
Effect of cooling condition on total symptom scores after controlling for cumulative effects of mean skin temperature. Responses were evaluated in older adults (69-74 y; *n* = 17) at the end of three randomized 6-h extreme heat exposures (38°C, 35% relative humidity). Conditions differed only in the applied cooling intervention: no cooling (control, blue), submersion of the feet to mid-calf in 20°C water for the last 40 min of each hour (foot immersion, gray), or foot immersion with a wet towel (20°C) draped around the neck (foot immersion with neck cooling, yellow). The red points and error bars represent the mean (95% confidence intervals) responses for each condition. Total symptom scores were derived from the 68-item environmental symptoms questionnaire, version 4 (ESQ-IV). End-heating symptom scores were evaluated using a negative binomial generalized linear mixed model with a log link function. Cooling condition (categorical predictor), as well as pre-exposure score (Score_pre; continuous predictor) and skin temperature area under the curve (Tskin_auc, hours 0-6; continuous predictor) were included as fixed effects. To account for repeated measurements due to the crossover design, a random intercept was modeled for each participant. Significance was set at *p* < 0.05 (two-sided).

There was a significant effect of cooling condition on total symptom score at end-heating (*p* = 0.013; Figure 1), which, when compared to the no-cooling control condition, was 0.78-fold [0.62, 0.99] lower during the foot immersion condition (*p* = 0.036) and 0.78-fold [0.61, 0.99] lower during the foot immersion with neck cooling condition (*p* = 0.036). There were no significant independent effects of any index of cumulative physiological strain on end-heating total symptom scores (all *p* ≥ 0.487). After adjusting for mean skin temperature AUC, the effect of cooling condition on total symptom score was no longer significant (*p* = 0.171; Figure 2). By contrast, the effect of cooling condition on total symptom score remained after adjusting for all other indices of physiological strain (all *p* ≤ 0.023; Supplemental Table 1).

**Figure 3.**
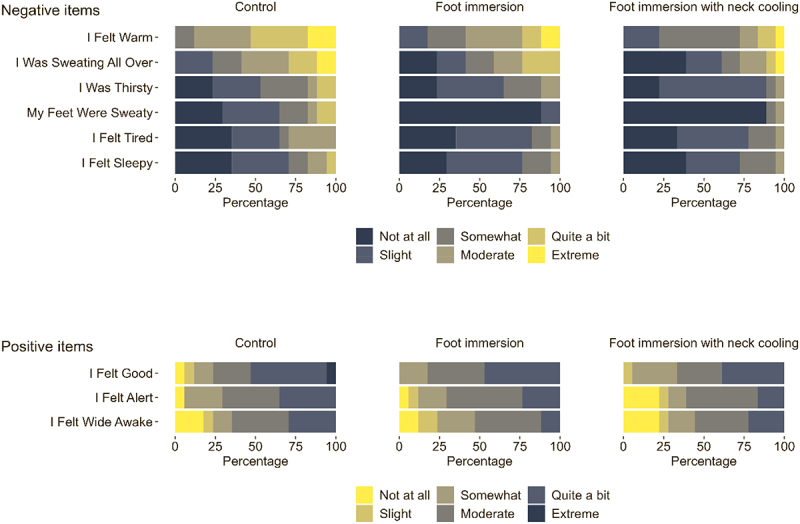
Rank order of individual item responses at end-heating from the 68-item environmental symptoms questionnaire, version 4 (ESQ-IV). Grouped raw score responses are reported for older adults (69-74 y; *n* = 17) at the end of three randomized 6-h extreme heat exposures (38°C, 35% relative humidity). Conditions differed only in the applied cooling intervention: no cooling (control), submersion of the feet to mid-calf in 20°C water for the last 40 min of each hour (foot immersion), or foot immersion with a wet towel (20°C) draped around the neck (foot immersion with neck cooling). For each item, participants were asked to rank their response based on a 6 level likert scale (0 = not at all, 1 = slight, 2 = somewhat, 3 = moderate, 4 = quite a bit, 5 = extreme). Only items with group mean ratings ≥ 1 (“slight”) are presented here, representing a minimum stimulus response. Individual items for all conditions were rank ordered by group mean, then by prevalence of responses for the no-cooling control condition.

The rank order of individual ESQ-IV item responses at end-heating is presented in Figure 3. For the no-cooling control condition, participants reported six negative items (symptoms) and three positive items with mean scores ≥1 [“Slight”], representing the minimum threshold stimulus response at the group level. Overall, foot immersion and foot immersion with neck cooling were associated with lower mean responses for the negative items (e.g. “I felt warm,” “I was sweating all over”) and comparable mean responses for each of the positive items (e.g. “I felt good,” “I felt alert”) compared to the no-cooling control condition.

### Mood disturbance

There were no statistically significant effects of cooling condition on total mood disturbance (*p* = 0.460; Figure 1) or energy index (*p* = 0.275; Figure 1). There were also no significant independent effects of any index of cumulative physiological strain on the end-heating total mood disturbance (all *p* ≥ 0.250) or energy index scores (all *p* ≥ 0.127). After adjusting for all indices of physiological strain, the effects of cooling condition were non-significant for mood disturbance (all *p* ≥ 0.403) and energy index (all *p* ≥ 0.271; Supplemental Table 1).

**Figure 4.**
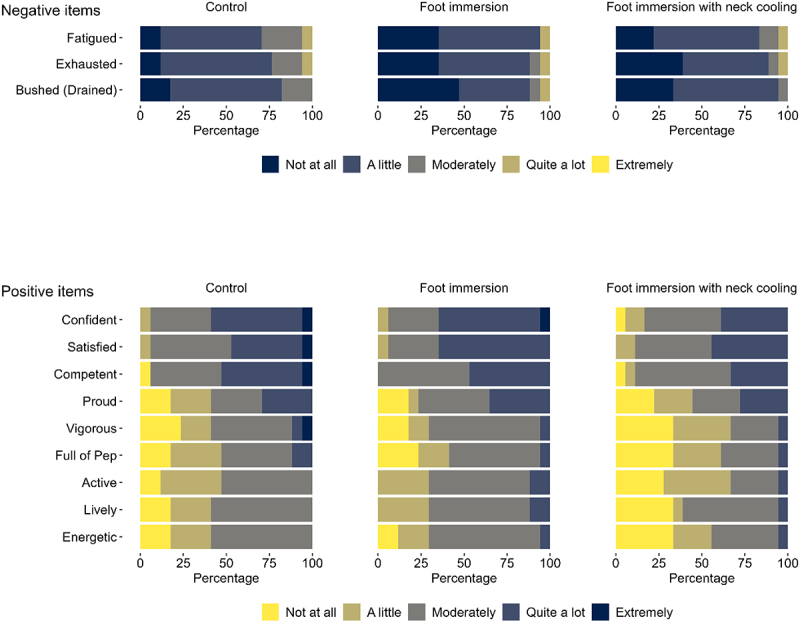
Rank order of individual item responses at end-heating from the 40-item profile of mood states questionnaire (POMS-40). Grouped raw score responses are reported for older adults (69-74 y; *n* = 17) at the end of three randomized 6-h extreme heat exposures (38°C, 35% relative humidity). Conditions differed only in the applied cooling intervention: no cooling (control), submersion of the feet to mid-calf in 20°C water for the last 40 min of each hour (foot immersion), or foot immersion with a wet towel (20°C) draped around the neck (foot immersion with neck cooling). For each item, participants were asked to rank their response based on a 5 level likert scale (0 = not at all, 1 = a little, 2 = moderately, 3 = quite a lot, 4 = extremely). Only items with group mean ratings ≥ 1 (“A little”) are presented here, representing a minimum stimulus response. Individual items for all conditions were rank ordered by group mean, then by prevalence of responses for the no-cooling control condition.

The rank order of individual POMS-40 item responses at end-heating is presented in Figure 4. For the no-cooling control condition, participants reported three negative items and nine positive items with mean scores ≥1 [“A little”], representing the minimum threshold stimulus response at the group level. Overall, foot immersion with and without neck cooling was associated with comparable mean responses for each of the negative and positive items compared to the no-cooling control condition.

### Body temperature and hemodynamic responses

**Figure 5.**
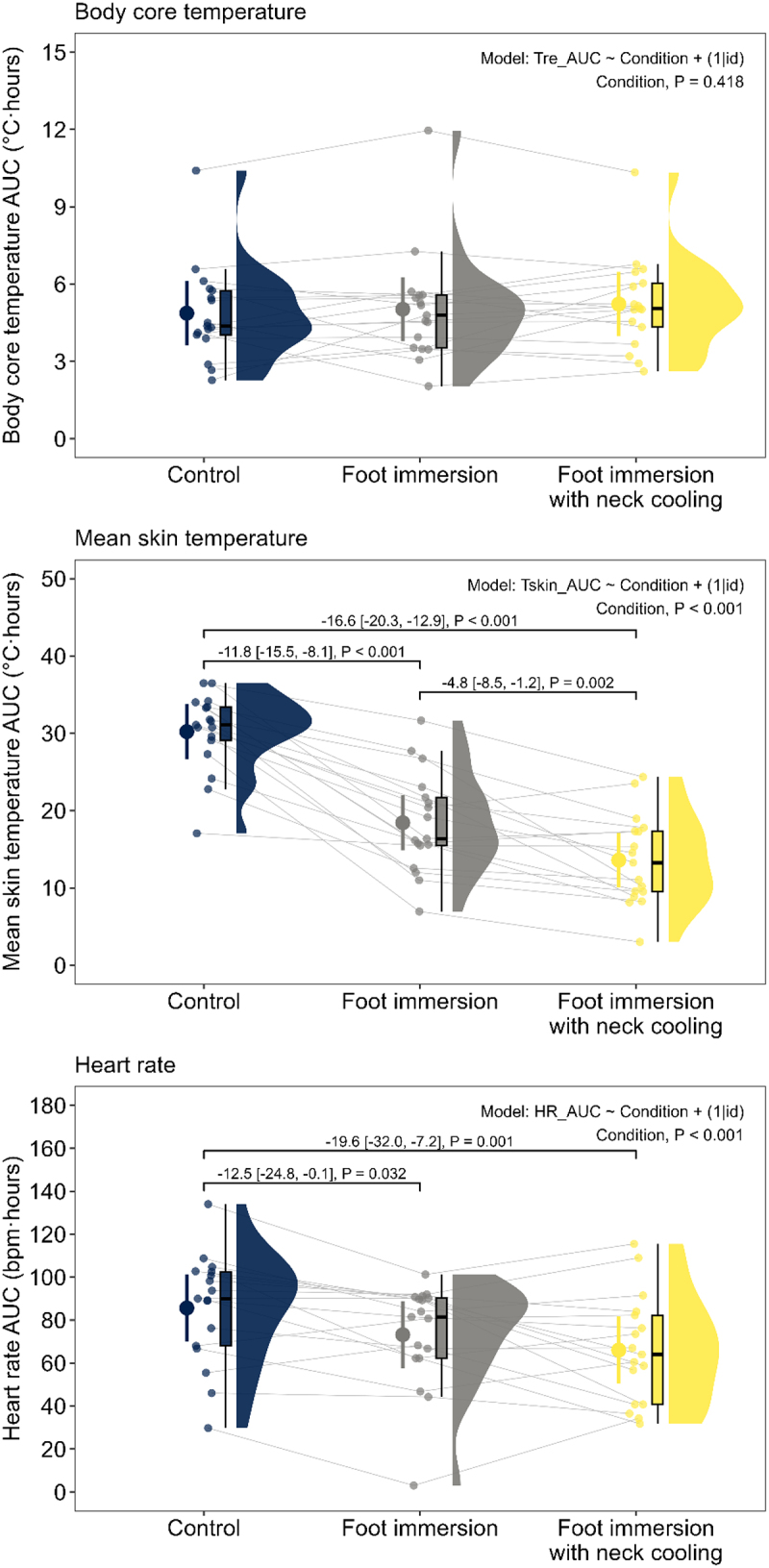
Influence of cooling condition on the physiological responses of older adults (69-74 y; n = 17) at the end of three randomized 6-h extreme heat exposures (38°C, 35% relative humidity). Conditions differed only in the applied cooling intervention: no cooling (control), submersion of the feet to mid-calf in 20°C water for the last 40 min of each hour (foot immersion), or foot immersion with a wet towel (20°C) draped around the neck (foot immersion with neck cooling). The cumulative physiological effects of each condition were evaluated by assessing the areas under the curve (AUC) for body core temperature (Tre, top panel), mean skin temperature (Tskin, middle panel), and heart rate (HR, lower panel). Data are presented as modeled means (large points) and 95% confidence intervals (error bars) with individual scores (small points), boxplots (median and interquartile range), and density plots (distribution) for each condition. Between-condition contrasts are presented as mean differences [95% confidence limits]. Significance was set at p < 0.05 (two-sided). *p* values were adjusted for multiplicity using the Holm-Bonferroni procedure.

There was no significant effect of cooling condition on body core temperature AUC (*p* = 0.418; Figure 5). By contrast, there was a significant effect of cooling condition on mean skin temperature AUC (*p* < 0.001; Figure 5), which was 11.8°C · h [95% confidence interval: 8.1, 15.5] lower in the foot immersion condition (*p* < 0.001) and 16.6°C · h [12.9, 20.3] lower in the foot immersion with neck cooling condition (*p* < 0.001), compared to the no-cooling control condition. Further, mean skin temperature AUC was 4.8°C · h [1.2, 8.5] lower in the foot immersion with neck cooling condition compared to the foot immersion alone condition (*p* = 0.002).

There was a significant effect of cooling condition on heart rate AUC (*p* < 0.001; Figure 5), which was 12.5 bpm · h [0.1, 24.8] lower in the foot immersion condition (*p* = 0.032) and 19.6 bpm · h [7.2, 32.0] lower in the foot immersion with neck cooling condition (*p* = 0.001), compared to the no-cooling control condition. By contrast, there were no significant effects of cooling condition on either systolic blood pressure AUC (*p* = 0.972) or diastolic blood pressure AUC (*p* = 0.573).

## Discussion

We found that immersing the feet in cool water, with or without the addition of a cool wet towel to the neck reduced skin temperature but had little effect on body core temperature or cardiovascular strain [13]. Notably, the reduction in skin temperature was associated with a reduction in self-reported symptoms of environmental stress, despite having no influence on mood-state across the 6-h exposure to a simulated overheated dwelling.

### Effect of limb immersion and neck cooling on environmental symptoms

Our primary objective was to assess the effect of foot immersion with and without neck cooling on total environmental symptom scores and mood state in older adults exposed to a simulated overheated dwelling, representive of conditions experienced in-homes during extreme heat events. We found that these interventions decreased skin temperature and total environmental symptom scores despite having a negligible effect on core temperature and only a small effect on cardiovascular responses [13,23]. Interestingly, between condition differences in symptom scores were abated when analyses were adjusted for skin temperature, suggesting that reductions in the latter drove those in the former (Figure 2). This finding is consistent with previous investigations [14,28,37]in which it was suggested that a reduction in skin temperature, be it transient or sustained, is a major contributing factor to self reported environmental symptom scores in older adults. For example, previous work from our laboratory has shown that exposure to a brief 2-h cooling period at the mid-point of a daylong (9 h) heat exposure reduced the total symptom score at the end of the heat exposure [14]. While the mid-day cooling intervention reduced both core and skin temperatures, only the latter were statistically associated with self-reported symptom scores [14]. Consistent with findings in young adults [37,38], we show that cumulative elevations in mean skin temperature have a greater influence on self-reported symptoms of heat illness in older adults than core temperature elevations. While previous studies [14,28], when taken together with our observations, indicate that cooling-induced reductions in skin temperature contribute to decreased total symptom scores at end-heating, it remains unclear whether a reduction in self-reported environmental symptoms may impact willingness to act or change behavior in the face of environmental heat stress. Indeed, taking appropriate action and changing behavior in the face of environmental heat stress is of critical importance for the health and safety of heat-vulnerable older adults, who have a decreased capacity to sense and respond to the dangers of heat stress [39]. Thus, while evidence suggests that skin temperature elevations are a major contributing factor to behavioral thermoregulation [37] and we show that skin temperature reductions decrease environmental symptoms, detailed examinations aimed at assessing the relation between skin temperature, total symptom scores, and willingness to change behavior (e.g. visit a cooling center, implement known cooling strategies) in isolation are needed.

### Effect of limb immersion and neck cooling on mood-state

Previous investigations have reported that cooling interventions (brief exposure to an air-conditioned environment) can limit the increase in negative mood state of older adults associated with prolonged exposure (9-h) to overheated indoor environments [14]. By contrast, however, we witnessed no association between limb immersion with or without neck cooling and change in mood state or energy index compared to the no-cooling control condition. A possible explanation for this could be a result of the lack of meaningful effect on core temperature and cardiovascular strain in the current investigation, compared to previous research [14]. Indeed, as highlighted above, data from our laboratory has previously shown that brief exposure to ambient cooling (2-h exposure to an air-conditioned environment) during the mid-point of a 9-h heat exposure (40°C and 9% RH, conditions consistent with peak indoor temperatures recorded during recent deadly heat events [5]) reduces cumulative thermal and cardiovascular strain in older adults after returning to the heat. Thus, in contrast to the current investigation, whereby foot immersion and neck cooling did not influence core temperature, and had little meaningful effect on cardiovascular strain (across the 6-h exposure period), exposure to an air-conditioned environment [14,40] can have a significant physiological effect that appears to improve mood-state at the completion of a 9-h heat exposure [14]. However, it is important to note that at the time of measurement in the aforementioned investigation [14], thermal and cardiovascular strain had already returned to precooling levels [14,40], indicating that despite a directive to describe “how do you feel right now” a prior reduction in physiological strain is a contributing factor to overall mood-state at a given point in time. Together, in conjunction with previous research [14,28,37,41–43], these findings highlight the complexities of how individuals perceive environmental heat stress, and how this subsequently influences mood-state. Delineating how these complexities influence thermal behavior is an important area for future work.

### Limitations

An important limitation of the current study is that we evaluated the effects of foot immersion with and without neck cooling on mood states and environmental symptom scores under a single set of ambient conditions (38°C, 35% relative humidity). Furthermore, we only evaluated perceptual responses at the end of the heat exposure period (i.e. 6-h). While these were necessary constraints due to the time- and resource-intensive nature of the trial, whether divergent results would have been witnessed when exposed to different ambient conditions or longer heating exposures (i.e. ≥8-h) more typical of a daylong overheated indoor environment remains unclear. Nonetheless, the findings of the current investigation further reinforce the importance of, where possible, maintaining ambient temperature within the home within safe and comfortable limits, and thus avoiding the known risks associated with prolonged exposure to elevated indoor temperatures [44], especially for vulnerable population groups [3].

## Conclusion

Our data show that foot immersion with and without neck cooling lowers self-reported total symptoms of environmental stress despite having little meaningful effect on body core temperature, heart rate responses and mood-state of older adults exposed to an indoor overheated environment for 6-h. While a reduction in self-reported environmental symptoms may appear to be of benefit, behavior change must be preceded by a perceived need to act. Therefore, a cooling intervention induced reduction in the perception of environmental stress, despite a lack of meaningful effect and core temperature and cardiovascular strain could negatively impact subsequent behavior, which may place heat-vulnerable older adults at greater risk for developing the adverse health effects of exposure to indoor overheating. Therefore, continued vigilance and the use of appropriate countermeasures (e.g. visiting cooling centers or air-conditioned spaces) to mitigate physiological strain during periods of extreme heat are required to protect the most vulnerable when in-home air-conditioning is not available. The findings presented within this investigation further our understanding of the effect of commonly recommended cooling interventions on the perception of environmental symptoms and mood-states of heat in vulnerable older adults exposed to indoor overheating.

## Supplementary Material

Limb POMS_ESQ supplement.docx

## Data sharing statement

Deidentified participant data are available from the corresponding author (gkenny@uottawa.ca) upon reasonable request and signed access agreement.

## List of abbreviations

AUC: area under the curve
bpm: beats per minute
EI: energy index
ESQ-IV: environmental symptoms questionnaire
IQR: interquartile range
mmHg: millimeters of mercury
POMS-40: profile of mood states
TMD: total mood disturbance
