# Supplementary material for: Foot immersion with and without neck cooling reduces self-reported environmental symptoms in older adults exposed to simulated indoor overheating
Source: Temperature (Austin). 2024 Sep 11;11(4):318–32. doi: 10.1080/23328940.2024.2394341 (PMC11583589; doi:10.1080/23328940.2024.2394341)
Supplement: Limb POMS_ESQ supplement.docx [file KTMP_A_2394341_SM0235.docx]

**Effects of foot immersion and neck cooling on self-reported symptoms and mood-state in older adults exposed to indoor overheating**

Fergus K. O’Connor^1^; Gregory W. McGarr^1,2^; Emma McCourt^1^; Robert D. Meade^1^; Glen P. Kenny^1,3^

**Affiliations**

^1^Human and Environmental Physiology Research Unit, School of Human Kinetics, Faculty of Health Sciences, University of Ottawa, Canada; ^2^Consumer and Clinical Radiation Protection Bureau, Health Canada, Ottawa, Ontario, Canada; ^3^Clinical Epidemiology Program, Ottawa Hospital Research Institute, Ottawa, Canada

Supplemental Results

**Table S1.** Effect of cooling condition on questionnaire responses at the end of the 6-hour exposure to extreme heat (38°C, 35% relative humidity) after controlling for physiological strain.

|  | **Raw data**, *mean (SE)* | | |  | **Foot immersion** ^a^ | |  | **Foot immersion**  **with neck cooling** ^a^ | | **Condition *P-value*** ^b^ | **Strain Index *P-value ^c^*** |
| --- | --- | --- | --- | --- | --- | --- | --- | --- | --- | --- | --- |
|  | Control | Foot immersion | Foot immersion with neck cooling |  | Mean [95% CI] | P-value |  | Mean [95% CI] | P-value |  |  |
| **Body core temperature AUC adjusted model** | | | |  |  |  |  |  |  |  |  |
| Total Symptom Score | 31 (3) | 24 (2) | 24 (2) |  | 0.78-fold [0.62, 0.98] | 0.028 |  | 0.77-fold  [0.61, 0.98] | 0.028 | 0.010 | 0.487 |
| Total Mood Disturbance | 84 (2) | 80 (2) | 82 (2) |  | 0.95-fold [0.87, 1.05] | 0.657 |  | 0.98-fold  [0.89, 1.07] | >0.999 | 0.470 | 0.269 |
| Energy Index | 2.1 (1.4) | 4.3 (1.4) | 2.6 (1.4) |  | 2.2 [-1.6, 5.9] | 0.443 |  | 0.5 [-3.2, 4.2] | 0.738 | 0.300 | 0.180 |
| **Mean skin temperature AUC adjusted model** | | | |  |  |  |  |  |  |  |  |
| Total Symptom Score | 32 (4) | 24 (2) | 24 (3) |  | 0.76-fold [0.53, 1.08] | 0.183 |  | 0.75-fold  [0.48, 1.16] | 0.224 | 0.171 | 0.787 |
| Total Mood Disturbance | 84 (3) | 80 (2) | 82 (3) |  | 0.95-fold [0.84, 1.08] | >0.999 |  | 0.97-fold  [0.84, 1.13] | >0.999 | 0.571 | 0.964 |
| Energy Index | 3.1 (2.0) | 4.1 (1.5) | 1.9 (1.8) |  | 1.0 [-4.6, 6.7] | >0.999 |  | -1.2 [-8.4, 6.0] | >0.999 | 0.350 | 0.444 |
| **Heart rate AUC adjusted model** | | | |  |  |  |  |  |  |  |  |
| Total Symptom Score | 31 (3) | 24 (2) | 24 (2) |  | 0.78-fold [0.61, 0.99] | 0.044 |  | 0.78-fold  [0.60, 1.00] | 0.044 | 0.023 | 0.960 |
| Total Mood Disturbance | 84 (2) | 80 (2) | 82 (2) |  | 0.95-fold [0.86, 1.04] | 0.539 |  | 0.97-fold  [0.88, 1.07] | 0.848 | 0.403 | 0.573 |
| Energy Index | 2.1 (1.5) | 4.4 (1.4) | 2.6 (1.5) |  | 2.2 [-1.7, 6.1] | 0.471 |  | 0.5 [-3.7, 4.8] | 0.757 | 0.296 | 0.900 |
| **Systolic arterial blood pressure AUC adjusted model** | | | |  |  |  |  |  |  |  |  |
| Total Symptom Score | 31 (3) | 24 (2) | 24 (2) |  | 0.78-fold [0.61, 0.99] | 0.036 |  | 0.78-fold  [0.61, 0.99] | 0.036 | 0.013 | 0.530 |
| Total Mood Disturbance | 84 (2) | 80 (2) | 82 (2) |  | 0.95-fold [0.87, 1.04] | 0.571 |  | 0.98-fold  [0.89, 1.07] | >0.999 | 0.424 | 0.250 |
| Energy Index | 2.0 (1.3) | 4.4 (1.4) | 2.7 (1.3) |  | 2.4 [-1.4, 6.2] | 0.374 |  | 0.7 [-3.1, 4.5] | 0.643 | 0.271 | 0.127 |
| **Diastolic arterial blood pressure AUC adjusted model** | | | |  |  |  |  |  |  |  |  |
| Total Symptom Score | 31 (2) | 24 (2) | 24 (2) |  | 0.78-fold [0.62, 0.99] | 0.036 |  | 0.77-fold  [0.61, 0.99] | 0.036 | 0.014 | 0.630 |
| Total Mood Disturbance | 84 (2) | 80 (2) | 82 (2) |  | 0.95-fold [0.87, 1.05] | 0.666 |  | 0.97-fold  [0.89, 1.07] | 0.949 | 0.469 | 0.254 |
| Energy Index | 2.0 (1.4) | 4.2 (1.4) | 2.8 (1.4) |  | 2.2 [-1.6, 6.0] | 0.445 |  | 0.8 [-3.0, 4.6] | 0.742 | 0.326 | 0.192 |

Values are marginal means and standard error (SE) and either fold difference (Total Symptom Score, Total Mood Disturbance) or mean difference (Energy Index) from the control condition and 95% confidence interval (CI)^a^. Data reported for n = 17. Total Symptom Score is derived from the 68-item Environmental Symptoms Questionnaire, version IV; Total Mood Disturbance and Energy Index are derived from the 40-item Profile of Mood States questionnaire (POMS-40).

Results are derived from a (generalized) linear mixed-effects model comparing end-exposure questionnaire responses between Conditions: no cooling (control), submersion of the feet to mid-calf in 20°C water for the last 40 min of each hour (foot immersion), or foot immersion with a wet towel (20°C) draped around the neck (foot immersion with neck cooling). Responses (Score_End) are adjusted for baseline values (Score_Pre) and each index of physiological strain (based on area under the curve (AUC) from hours 0-6); PhysiolStrain_AUC). To account for repeated measurements due to the crossover design, a random intercept was modeled for each participant (id). Model: Score_End ~ Score_Pre + Condition + PhysiolStrain_AUC + (1|id). Confidence intervals and P-values are adjusted for multiplicity using the Holm-Bonferroni procedure (all comparisons for each variable were treated as a family of comparisons).

^b^ Analysis of variance P-value for the effect of condition from the linear mixed-effects model.

^c^ Analysis of variance P-value for the effect of the physiological strain index (AUC) from the linear mixed-effects model.
